# Determination of the Kinetic Rates and Intrinsic Nonradiative Losses in Organic Thermally Activated Delayed Fluorescence Emitters

**DOI:** 10.1002/advs.202505338

**Published:** 2025-06-26

**Authors:** Xin Zhou, Jawid Nikan, Kaiwen Guo, Paul W. M. Blom, Gert‐Jan A. H. Wetzelaer, Yungui Li

**Affiliations:** ^1^ Max Planck Institute for Polymer Research Ackermannweg 10 55128 Mainz Germany

**Keywords:** exciton dynamics, nonradiative loss, organic light‐emitting diodes, thermally activated delayed fluorescence, transient photoluminescence

## Abstract

Knowledge of kinetic rates of intersystem crossing (ISC) *k*_ISC_, reverse ISC *k*_rISC_, singlet *k*_S_, and triplet *k*_T_ relaxation processes, respectively, is indispensable for material design as well as understanding of the operation of organic light‐emitting diodes (OLEDs) based on organic emitters with thermally activated delayed fluorescence (TADF). Typically, the various rate constants are obtained by numerically fitting of the photoluminescence (PL) decay, but it is overlooked whether or not the solution of such a fit is unique. Using an analytical model, it is demonstrated that for typical TADF emitters only the sum of *k*_S_ and *k*_ISC_, and the sum of *k*_T_ and *k*_rISC_ can be uniquely obtained from the transient PL decay and PL quantum yield (PLQY) experiments. Depending whether nonradiative losses stem from singlets or triplets, various combinations of *k*_T_ and *k*_rISC_, *k*_S_ and *k*_ISC_ can be used to obtain the same transient PL decay, while keeping the PLQY constant. The only exception is the case of unity PLQY that allows for a direct determination of all relevant kinetic rates from just a single transient PL measurement. It is further shown that additional PL measurements in oxygen atmosphere with complete triplet quenching are redundant, since the results can be explicitly predicted from PL measurements in inert atmosphere. Using the TADF emitter CzDBA with a PLQY as high as 90% as a model system, it is demonstrated that by a combination of PL decay, PLQY, and OLED device modeling it is possible to quantify all the individual kinetic rates. For CzDBA neat film with PLQY as high as 90%, the majority of nonradiative losses stems from triplets, showing it is vital to conduct comprehensive studies on the molecular origin of nonradiative losses in the future.

## Graphical abstract

It is demonstrated that the kinetic rates and intrinsic nonradiative losses of purely organic emitters with thermally activated delayed fluorescence cannot be precisely determined by photoluminescent measurements alone. With device modeling, it is then possible to quantify the absolute nonradiative losses from singlets and triplets, and therefore the kinetic rates.

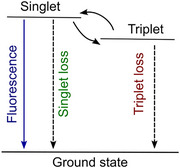

## 1 Introduction

Organic emitters exhibiting thermally activated delayed fluorescence (TADF) are considered promising candidates for the next generation of organic light‐emitting diodes (OLEDs) because of their capability to harvest triplet excitons at room temperature without the use of heavy atoms.^[^ ^1^, ^2^ ^]^ In OLEDs, injected holes and electrons recombine to generate both singlet and triplet excitons in a 1:3 ratio.^[^ ^3^, ^4^ ^]^ In conventional fluorescent emitters, due to low spin‐orbit coupling, the generated triplets relax nonradiatively, resulting in a low charge‐to‐photon efficiency *η*_CTP_ with a maximum of 25%.^[^ ^3^, ^5^ ^]^ TADF emitters can convert nonradiative triplets through reverse intersystem crossing (rISC) to radiative singlets, giving rise to delayed fluorescence (DF) in a transient decay.^[^ ^6^ ^]^ Therefore, unity charge‐to‐photon efficiency can be achieved in OLEDs based on highly efficient TADF emitters.

Because of the intersystem crossing (ISC) and rISC processes, the photoluminescence (PL) decay of TADF materials consists of prompt fluorescence (PF) and delayed fluorescence. Compared with the PF from direct singlet radiative decay in the nanosecond regime, the DF has a much longer transient lifetime, in the microsecond regime, due to rISC‐ISC cycling. The transient PL decay of a TADF emitter is determined by the ISC and rISC interconversion rates, as well as the singlet and triplet exciton decay rates. These kinetic rates play a central role in the efficiency, roll‐off, and the operational stability of TADF‐based OLEDs.^[^ ^7^, ^8^, ^9^ ^]^ Therefore, a comprehensive understanding of the transient PL decay of TADF emitters is of fundamental importance for the exciton dynamics and its relation to the OLED performance.

The rate constants of the various kinetic processes are typically obtained by modeling of the transient PL decay in combination with measurement of the photoluminescence quantum yield (PLQY).^[^ ^10^, ^11^ ^]^ Furthermore, to evaluate nonradiative contributions, it was proposed to additionally take the PLQY in oxygen into account to quench triplet excitons.^[^ ^10^ ^]^ As an alternative approach, it has been proposed to estimate the quantum yields of PF and DF from transient PL decays, from which the individual kinetic rates of TADF emitters can be determined under the assumption that nonradiative losses merely stem from singlets.^[^ ^12^ ^]^ However, a fundamental question is how the determination of the rate constants is affected when nonradiative losses do not only arise from singlets but also from triplets.^[^ ^13^, ^14^, ^15^ ^]^ Nonradiative decay of triplets is especially essential for the OLED efficiency, since in an OLED, due to spin statistics, more triplets are generated as compared to a PL decay measurement.^[^ ^16^ ^]^ Another important question is therefore whether a combination of PL measurements in both inert atmosphere and in oxygen with complete triplet quenching is sufficient for a unique quantification of triplet nonradiative losses.

In this work, we conduct a systematic and comprehensive study on the analysis of the PL decay of TADF emitters. The paper is organized as follows: In the first section, we demonstrate that for TADF systems with PLQY close to unity, the kinetic rates of exciton dynamics can be obtained analytically with the biexponential fitting parameters of the transient PL decay. In the second section, we discuss the more common case of TADF emitters with nonradiative losses, in which the PLQY is lower than 100%. We show that different combinations of kinetic rates can be used to obtain the same PL decay and PLQY. We then proceed to demonstrate that the lack of a unique solution cannot be resolved by additional PL measurements in oxygen, since the PL decay and PLQY with complete triplet quenching can be explicitly predicted from PL measurements done in nitrogen. In the last section, we show that a combination of the transient PL decay, PLQY, and the charge‐to‐photon efficiency obtained from OLED analysis enables quantification of the nonradiative singlet and triplet losses and therefore all the individual kinetic rates of the excitonic processes.

## 2 Results

### 2.1 Rate Determination from Transient PL Decays for TADF Emitters with PLQY Close to Unity

For TADF emitters, the triplet relaxation is nonradiative, such that the transient PL decay after the excitation pulse is determined by the singlet population as a function of time. Without bimolecular annihilation processes, i.e., at low excitation dose or in a highly diluted degassed solution, the transient PL decay is then only determined by the intrinsic kinetic rates of exciton dynamics, which are the ISC rate *k*_ISC_, the rISC rate *k*_rISC_, the nonradiative triplet decay rate *k*_T_, and the singlet relaxation rate *k*_S_, which is composed of the radiative rate $k_{r}^{S}$ and nonradiative rate $k_{\mathrm{nr}}^{S}$. The singlet and triplet evolution in the three‐level model can be describe by rate equations Equation S1 (Supporting Information), which has been widely used for numerically modeling the exciton dynamics of TADF emitters.^[^ ^11^, ^16^ ^]^

Experimentally, the max‐normalized PL decay in an inert atmosphere is typically fitted with a biexponential function A_1_exp(‐t/τ_PF_)+A_2_exp(‐t/τ_DF_), with A_1_ + A_2_ = 1. Such a transient PL behavior has been widely observed for many TADF emitters at room temperature and is a characteristic of the three‐level model.^[^ ^12^ ^]^ We note that more complicated models with multiple triplet states might be needed to describe the transient PL decays at lower temperatures.^[^ ^17^, ^18^, ^19^, ^20^, ^21^ ^]^ In this study we focus on the exciton dynamics of TADF emitters at room temperature. The occurrence of a biexponential decay has the large advantage that there is a direct relation between the intrinsic kinetic rates (i.e., Equation S2, Supporting Information) and the biexponential fitting parameters:^[^ ^16^ ^]^

(1) $$k_{\mathrm{rISC}}+k_{T}=\frac{A_{2}}{\tau_{\mathrm{PF}}}+\frac{A_{1}}{\tau_{\mathrm{DF}}}$$

(2) $$k_{r}^{S}=\frac{\eta_{\mathrm{PLQY}}}{A_{1}\tau_{\mathrm{PF}}+A_{2}\tau_{\mathrm{DF}}}\;$$

(3) $$k_{S}+k_{\mathrm{ISC}}=\frac{A_{1}}{\tau_{\mathrm{PF}}}+\frac{A_{2}}{\tau_{\mathrm{DF}}}$$

here, the photoluminescence quantum yield *η*_PLQY_ in an inert atmosphere is independently measured.

For highly efficient TADF systems with unity PLQY, nonradiative losses from singlets or triplets are absent. In that case, since *k_s_* =$k_{r}^{S}$ and *η*_PLQY_ = 1, Equations (1), (2), (3) simplifies to:

(4) $$k_{\mathrm{rISC}}=\frac{A_{2}}{\tau_{\mathrm{PF}}}+\frac{A_{1}}{\tau_{\mathrm{DF}}}$$

(5) $$k_{S}=\frac{1}{A_{1}\tau_{\mathrm{PF}}+A_{2}\tau_{\mathrm{DF}}}\;$$

(6) $$k_{\mathrm{ISC}}=\frac{A_{1}}{\tau_{\mathrm{PF}}}+\frac{A_{2}}{\tau_{\mathrm{DF}}}-\frac{1}{A_{1}\tau_{\mathrm{PF}}+A_{2}\tau_{\mathrm{DF}}}$$

**Figure 1:**
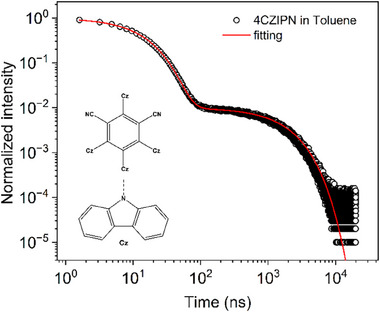
Determination of kinetic rates from the transient PL decay. The transient PL decay for 4CzIPN in degassed toluene (20 µm) was measured by a TCSPC setup (PicoQuant), excited with a ps‐laser at 375 nm with a repetition rate of 50 kHz. The monitored wavelength was 550 nm. Inset: chemical structure of 4CzIPN.

In other words, for highly efficient TADF systems it is possible to determine the individual kinetic rates just from a biexponential fit of the PL decay alone, without the need for numerical modeling or temperature‐dependent transient PL measurements. We illustrate this concept for the TADF emitter 4CzIPN (1,2,3,5‐tetrakis(carbazol‐9‐yl)‐4,6‐dicyanobenzene,2,4,5,6‐tetrakis(9H‐carbazol‐9‐yl) isophthalonitrile).^[^ ^1^ ^]^ Its chemical structure is shown as an inset in **Figure** **1**. In degassed toluene, the absolute PLQY of 4CzIPN is as high as 94.0%.^[^ ^1^ ^]^ The kinetic rates in degassed toluene have been characterized previously by Adachi's group by temperature‐dependent transient PL decays, with the assumption that singlets contribute to the nonradiative loss.^[^ ^12^ ^]^

The transient PL decay of 4CzIPN in degassed toluene at room temperature is measured by time‐correlated single‐photon counting (TCSPC) with a low‐power diode ps‐laser. In a highly diluted degassed solution with a concentration as low as 20 µm, singlet‐triplet and triplet‐triplet annihilation, as well as oxygen quenching is avoided. As shown in Figure 1, the transient PL decay is well described by a biexponential function, with the fitting parameters summarized in **Table** **1**.

**Table 1:** Photophysical rates for 4CzIPN in degassed toluene measured by different methods.

*η*_PLQY_	A_1_	A_2_	τ_PF_ [ns]	τ_DF_ [µs]	*k*_S_ [s^−1^]	*k*_ISC_ [s^−1^]	*k*_rISC_ [s^−1^]	$k_{r}^{S}$ [s^−1^]	$k_{\mathrm{nr}}^{S}$ [s^−1^]
94.0%	0.99	0.01	14.2	1.8	3.12 × 10^7^	3.85 × 10^7^	1.25 × 10^6^	2.94 × 10^7^	1.8 × 10^6^
94.0%			14.2	1.8	1.75 × 10^7^	5.1 × 10^7^	2.7 × 10^6^	1.7 × 10^7^	1.5 × 10^6^

^*^Rates in the first row are calculated from Equations (4)–(6), while that in the second row are taken from Ref. [12] for comparison.

Using Equations (4)–(6), the kinetic rates (*k*_S_, *k*_ISC_, *k*_rISC_) are then directly determined from the biexponential fit, as shown in Table 1 (first row). To compare with the reported method with the assumption that only singlets contribute to the nonradiative loss,^[^ ^12^ ^]^ we can further estimate the radiative and nonradiative singlets rates by *η*_PLQY_ = $k_{r}^{S}$/*k*_S_, and $k_{\mathrm{nr}}^{S}$ = *k*_S_‐$k_{r}^{S}$. As a comparison, the rates reported previously are also shown in Table 1 as the second row. Kinetic rates obtained by the current method compare to that determined from temperature‐dependent transient PL decays within a factor of two.^[^ ^12^ ^]^ The main discrepancy arises from a slight difference in the experimental delayed quantum yields. Furthermore, the value of the RISC rate was calculated from the singlet‐triplet splitting, obtained from temperature‐dependent measurements. Here, it is demonstrated that one can reliably obtain the kinetic rates from only a single transient PL measurement at room temperature and directly using the biexponential fitting parameters.

### 2.2 Transient PL Decay for TADF Systems with PLQY Lower Than 100%

In most of the practical cases, the PLQY of TADF emitters is lower than 100%. For example, it has been reported that the host material, or the doping concentration has a huge impact on the PLQY and exciton dynamics of TADF emitters.^[^ ^22^, ^23^ ^]^ Furthermore, the presence of pronounced nonradiative loss has been widely observed in orange, red, or NIR TADF emitters, where the energy gap law plays an important role.^[^ ^24^, ^25^ ^]^ Therefore, it is of general importance to analyze the situation in which nonradiative decay is present. In this section, we will demonstrate that the kinetic rates determined for TADF emitters based on the transient PL decay and PLQY are not unique. This is not a result of fitting uncertainty, but an intrinsic limitation, since different combinations of kinetic parameters can give rise to the same PL decay together with a fixed PLQY. We evaluate this fundamental problem using the well‐studied TADF emitter 5,10‐bis(4‐(9H‐carbazol‐9‐yl)‐2,6‐dimethylphenyl)‐5,10‐dihydroboranthrene (CzDBA).^[^ ^7^, ^26^, ^27^, ^28^, ^29^, ^30^, ^31^, ^32^ ^]^ The chemical structure of CzDBA is shown in Figure S2 (Supporting Information).

**Figure 2:**
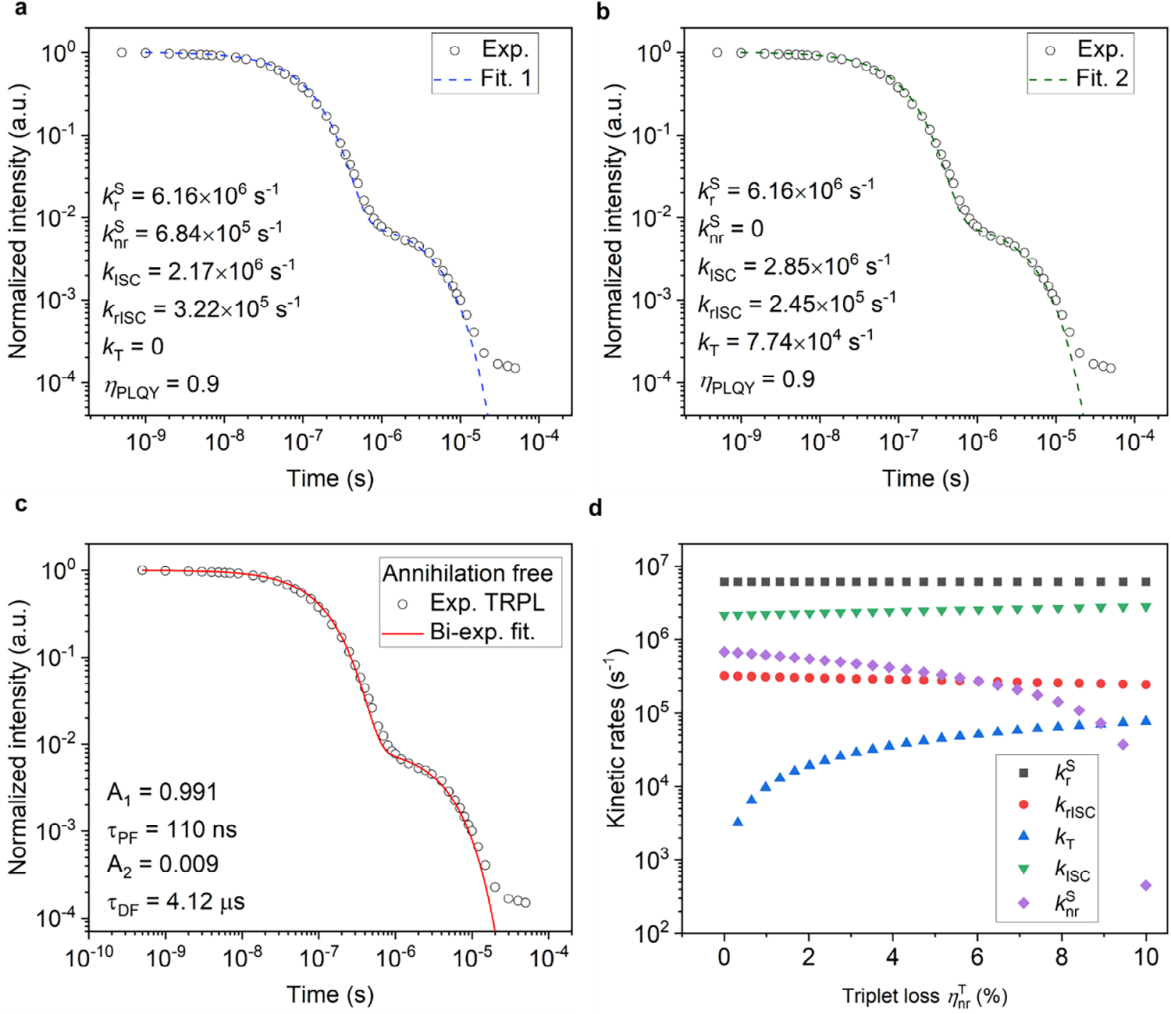
Systematic uncertainties in rate determination from the transient PL decay for TADF emitters with nonradiative losses. The transient PL decay was measured by a ps‐laser at 354 nm, under the intensity of 0.6 µW mm^−2^ with repetition rate of 10 kHz. The gated photoluminescence spectra and intensity was recorded by a 4Picos gated‐iCCD camera (Stanford Computer Optics). Here, the fitted curve described as the singlet evolution is obtained by numerically solving Equation S1 (Supporting Information) with the assumed kinetic parameters in (a) and (b). c) Biexponential fitting of the transient PL decay. d) The possible combinations of kinetic rates.

First, the transient PL decay of a CzDBA neat film is experimentally determined. To avoid the impact of exciton annihilation, the transient PL decay of CzDBA neat film was measured at low excitation intensities, with an initial singlet density as low as 1–2 × 10^20^ m^−3^. When further reducing the excitation intensity, identical transient PL decays are observed, confirming that annihilation processes do not play a role. The annihilation‐free transient PL decay is shown in **Figure** **2**. The PLQY of a CzDBA neat film is 90%, which has been independently determined.^[^ ^31^ ^]^

In Figure 2a,b, the transient PL decay is numerically fitted with two different sets of kinetic parameters. As shown in Figure S1 (Supporting Information), the PL spectra shift for the CzDBA neat film as a function of the decay time is minor. Therefore, excimer emission is minor in the benchmark CzDBA system. With the fitting parameters shown in Figure 2a, the nonradiative decay from singlets is dominating the nonradiative losses, without the nonradiative loss from triplets (*k*_T_ = 0). In the other case shown in Figure 2b, the nonradiative decay from triplets is dominating the nonradiative loss (Equation S3b, Supporting Information), since the nonradiative rate of singlets is zero. However, with these two different sets of parameters, the same PLQY is maintained, intrinsically described by the kinetic rates as Equation S3a (Supporting Information). This example demonstrates that for TADF emitters with nonradiative losses, it is possible to use different combinations of kinetic rates to fit the transient PL decay while maintaining a fixed PLQY. The values of the extracted parameters thus depend on the assumed origin of the nonradiative losses.

In the above example, the two extreme cases of nonradiative losses being either fully singlet or triplet dominated were discussed. To further evaluate the impact of a simultaneous occurrence of nonradiative singlet and triplet losses we can define a ratio parameter *α* as:

(7) $$\alpha =\frac{k_{\mathrm{rISC}}}{k_{\mathrm{rISC}}+k_{T}}\;$$

The parameter *α* represents the competition between the rISC process and triplet nonradiative losses. When the losses are fully singlet related *k_T_* = 0 and *α* = 1, whereas α«1 indicates a dominating contribution from nonradiative triplet losses. Using this definition for *α* it can be shown that

(8) $$k_{\mathrm{ISC}}=\frac{1}{\alpha }\;\frac{\frac{\tau_{\mathrm{PF}}}{\tau_{\mathrm{DF}}}{\left(\frac{\tau_{\mathrm{DF}}}{\tau_{\mathrm{PF}}}-1\right)}^{2}}{\frac{\tau_{\mathrm{DF}}}{A1}+\frac{\tau_{\mathrm{PF}}}{A2}}$$

(9) $$k_{\mathrm{rISC}}=\alpha \left(\frac{A_{2}}{\tau_{\mathrm{PF}}}+\frac{A_{1}}{\tau_{\mathrm{DF}}}\right)$$

from which also expressions for the other relevant parameters (*k*_S_, $k_{\mathrm{nr}}^{S}$, *k*_T_ and $\eta_{\mathrm{nr}}^{T}$) of exciton dynamics can be obtained (Equation S4, Supporting Information). This enables us to analytically investigate how the origin of nonradiative losses, either from triplets or singlets, affects the determination of the kinetic rates based on the transient PL measurements.

The fitting parameters for the PL decay of a CzDBA neat film with a biexponential function are shown in Figure 2c. In Figure 2d the obtained kinetic rate constants are plotted as function of the non‐radiative triplet losses $\eta_{\mathrm{nr}}^{T}$. Since the PLQY amounts to 90%, the total assumed nonradiative triplet losses range from 0 to 10%, with 0 indicating that all losses are singlet related (Figure 2b), whereas 10% means all losses arise from triplets (Figure 2a). Figure 2d graphically depicts that for TADF emitters with nonradiative losses the individual kinetic rates cannot be uniquely determined. Furthermore, together with Equation (1) it shows that one can only determine the kinetic rate $k_{r}^{S}$, the sum of *k*_S_ and *k*_ISC_, and the sum of *k*_T_ and *k*_rISC_ from the transient PL decay and PLQY experiments alone. Various combinations of *k*_T_ and *k*_rISC_, *k*_S_ and *k*_ISC_ with different nonradiative losses from triplets can be used to obtain the same transient PL decay, while keeping the PLQY as 90%, as shown in Figure 2d.

The reason that it is impossible to determine the nonradiative kinetic rates by transient PL and PLQY measurements is because of their internal correlation with the kinetic parameters, irrelevant to the experimental uncertainties. In other words, no matter how accurate the measurements and fittings can be, it is still not possible to precisely determine the intrinsic nonradiative losses from singlets and triplets. A question now remains what additional experiments need to be carried out in order to obtain the nonradiative triplet losses, thereby fixing the other kinetic rates.

### 2.3 PL Measurements with Complete Triplet Quenching is Redundant

In the previous section, we have shown that it is impossible to accurately obtain the kinetic rates by fitting the transient PL decay and PLQY measured in an inert atmosphere without information on the nonradiative losses. To account for this, it has been previously reported that combining transient PL experiments and the PLQY measured in oxygen assuming fully quenched triplets, can be used to quantify the loss from triplet decay.^[^ ^10^ ^]^ In the following, we demonstrate that the additional PL measurements with complete triplet quenching cannot disentangle the loss from singlets and triplets, because the PL decay and PLQY for TADF emitters with perfect triplet quenching can be explicitly predicted by the PL measurements done in an inert atmosphere.

#### 2.3.1 Theoretical Derivation

**Figure 3:**
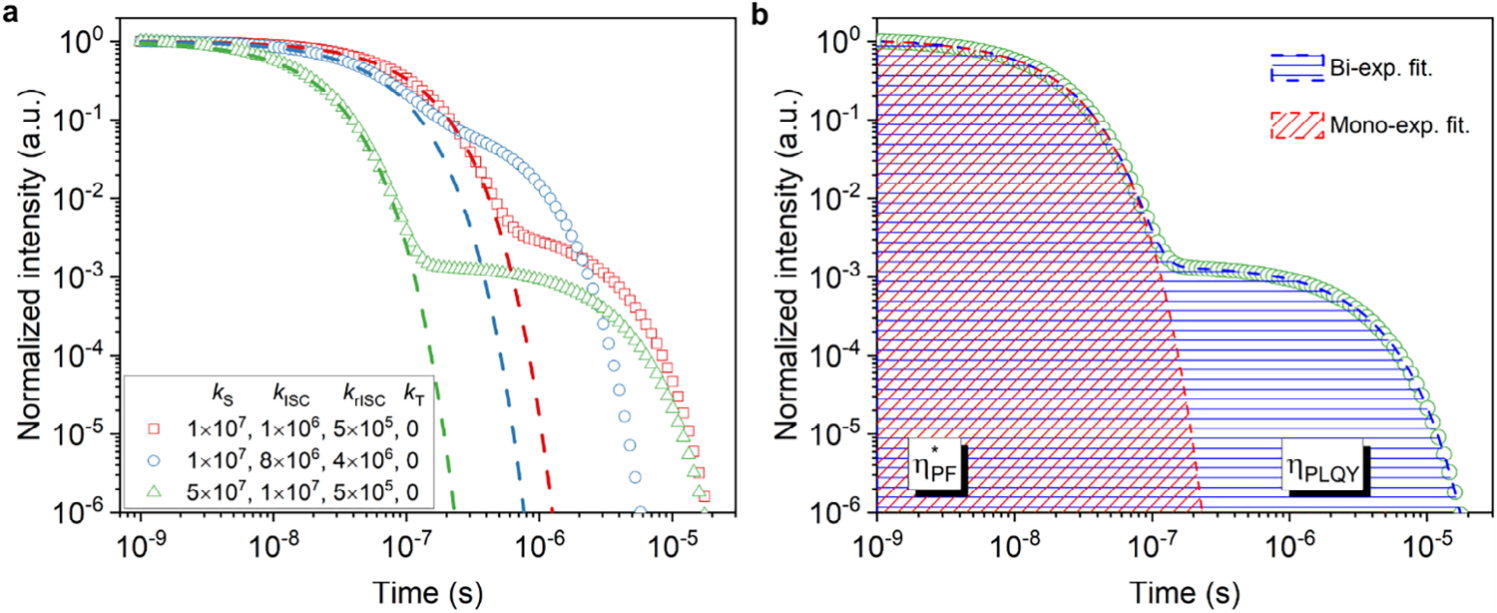
The determination of PF and DF from the max‐normalized PL decay. a) PL decay in inert atmosphere (symbols) as well as PL decay with perfect triplet quenching (dashed lines) are obtained by numerically modeling the evolution of singlet density (Equation S1, Supporting Information). The assumed kinetic rates (in s^−1^) used are indicated in the legend. b) Direct determination of the ratio of $\eta_{\mathrm{PF}}^{*}/\eta_{\mathrm{PLQY}}$ from the PL decays in an inert atmosphere.

**Figure** **3** displays numerically simulated PL decays for a few different sets of kinetic rates in an inert atmosphere (symbols), by solving the rate equations given by Equation S1 (Supporting Information). In addition, PL decays with complete triplet quenching are simulated (dashed lines). As the rISC process does not take place, only the PF remains, which is manifested as a monoexponential decay with a lifetime $\tau_{\mathrm{PF}}^{*}$ that coincides with the prompt part of the decay trace of the biexponential PL decay in an inert atmosphere.

When assuming efficient and complete triplet quenching in oxygen‐rich atmosphere without photo‐oxidization, the nonradiative process of triplet energy transfer dominates the triplet decay, superseding the rISC process. In such a case, the PL decay is only determined by the singlet decay including the spontaneous relaxation to the ground state with a rate of *k*_S_ and the ISC process with a rate of *k*_ISC_, which can be described by Equation S6a (Supporting Information). Therefore, the PLQY and the lifetime of the residual PL can be analytically described by the intrinsic kinetic rates, as shown in Equation S6c,d (Supporting Information). Furthermore, the PLQY of the residual fluorescence in case of complete triplet quenching, $\eta_{\mathrm{PF}}^{*}$ can also be expressed in the biexponential fitting parameters and *η*_PLQY_ measured in an inert atmosphere

(10) $$\eta_{\mathrm{PF}}^{*}=\frac{\eta_{\mathrm{PLQY}}}{A_{1}A_{2}\frac{\tau_{\mathrm{PF}}}{\tau_{\mathrm{DF}}}{\left(\frac{\tau_{\mathrm{DF}}}{\tau_{\mathrm{PF}}}-1\right)}^{2}+1}\;$$

Moreover, the transient PL lifetime $\tau_{\mathrm{PF}}^{*}$ with complete triplet quenching can be predicted from the biexponential fitting parameters in nitrogen as well:

(11) $$\frac{1}{\tau_{\mathrm{PF}}^{*}}=k_{S}+k_{\mathrm{ISC}}=\frac{A_{1}}{\tau_{\mathrm{PF}}}+\frac{A_{2}}{\tau_{\mathrm{DF}}}$$

As shown in Figure S3 (Supporting Information) and discussed in Supporting Information, $\tau_{\mathrm{PF}}^{*}$ exactly corresponds to the prompt PL lifetime, both in inert atmosphere and with triplet quenching. Since $\tau_{\mathrm{PF}}^{*}$ can be readily obtained in inert atmosphere, measurements in oxygen are redundant. Equations (10 and 11) give exact expressions for the $\eta_{\mathrm{PF}}^{*}$ and $\tau_{\mathrm{PF}}^{*}$ of residual PL for TADF systems in a triplet quenching atmosphere (e.g., oxygen) obtained only from the experimental PLQY and biexponential fitting parameters of the transient PL decay in an inert atmosphere. Therefore, one can precisely predict the remaining quantum yield and the decay lifetime under triplet quenching, based solely on the PLQY and transient PL decay in the inert atmosphere (e.g., nitrogen).

Furthermore, combining Equations (1), (2), (3) and Equations 10 and 11, the ratio between the residual PLQY $\eta_{\mathrm{PF}}^{*}$ with perfect triplet quenching and that in inert atmosphere can be written as:

(12) $$\frac{\eta_{\mathrm{PF}}^{*}}{\eta_{\mathrm{PLQY}}}=\frac{\tau_{\mathrm{PF}}^{*}}{A_{1}\tau_{\mathrm{PF}}+A_{2}\tau_{\mathrm{DF}}}\;$$

The ratio between $\eta_{\mathrm{PF}}^{*}$ and *η*_PLQY_, given by Equation 12, is graphically represented in Figure 3b. In the max‐normalized case of PL decays, with a biexponential fit A_1_exp(‐t/τ_PF_)+A_2_exp(‐t/τ_DF_), and (A_1_+A_2_ = 1), the *η*_PLQY_ can be calculated by the total integral of the transient PL trace over the entire time scale, which equals (*A*_1_τ_PF_+A_2_τ_DF_). The integrated area under the monoexponential fit of the PF decay in inert atmosphere then represents the PLQY with perfect triplet quenching, which equals $\tau_{\mathrm{PF}}^{*}$. Thus, the ratio of the PF area to the entire transient PL area in an inert atmosphere explicitly defines the ratio $\eta_{\mathrm{PF}}^{*}/\eta_{\mathrm{PLQY}}$. This analytical analysis rationalizes the commonly used method of integrating the underlying area of PF to determine PF and DF quantum yields.^[^ ^33^, ^34^ ^]^

#### 2.3.2 Experimental Verification

**Figure 4:**
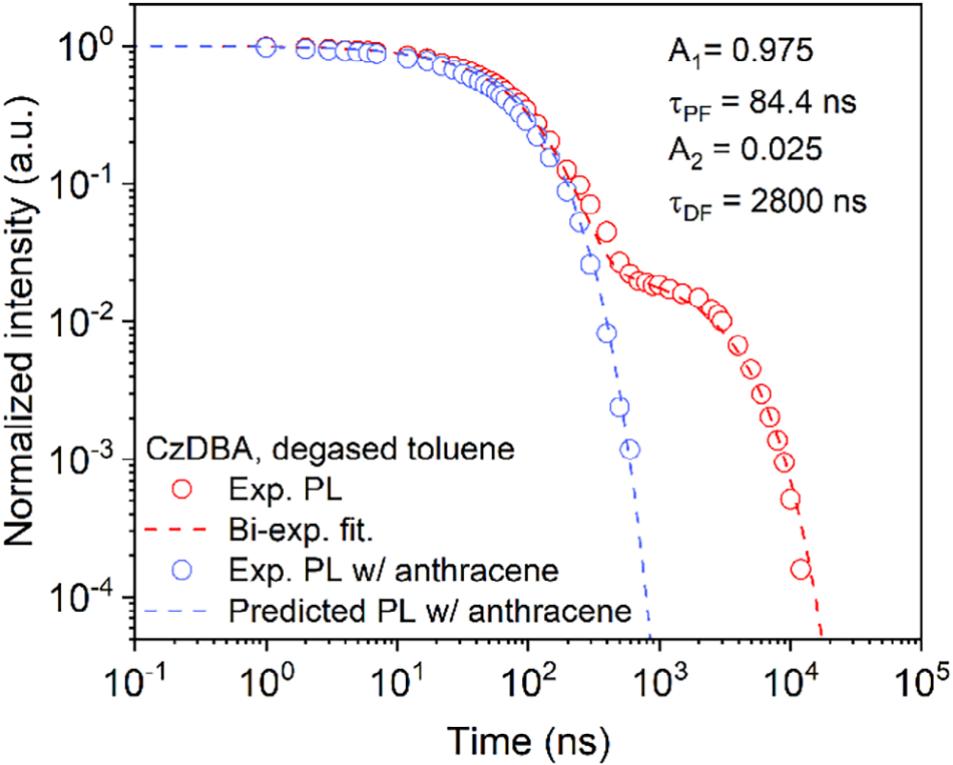
Transient PL decay of CzDBA in degassed toluene with (blue symbols) or without (red symbols) triplet scavenger anthracene. The highly diluted CzDBA solution at a concentration of 0.05 mg/ml is excited with an fs‐pulsed‐laser at 400 nm with a repetition rate of 1 KHz and a power density of 20 µW (Astrella, Coherent). The gated photoluminescence spectra and intensity is recorded by a 4Picos gated‐iCCD camera (Stanford Computer Optics). The biexponential fitting parameters for the transient decay without anthracene is shown as inset. The blue dashed line is the transient PL decay with perfect triplet quenching predicted from the biexponential fitting parameters.

We verify the above derivation with experiments on a CzDBA solution. As shown in **Figure** **4**, the experimental transient PL decay of CzDBA in degassed toluene can be well fitted with a biexponential function, with A_1_ = 0.975, τ_PF_ = 84.4 ns, A_2_ = 0.025 and τ_DF_ = 2.8 µs. Furthermore, the PLQY of CzDBA in degassed toluene is 25% and 13% in toluene with oxygen, respectively, which is experimentally determined by a calibrated integrating sphere.^[^ ^35^ ^]^

Using Equation 10, for $\eta_{\mathrm{PF}}^{*}$ a value of 14% is predicted from the PL measurements in degassed solution, which, considering the statistical uncertainties during PLQY measurements,^[^ ^36^ ^]^ is very close to the experimentally measured 13%.

To verify the theoretical calculations of the fluorescence decay trace (i.e., Equation 11 and Equation S6a, Supporting Information) in the presence of complete triplet quenching, we performed transient PL measurements with the addition of anthracene as a triplet scavenger. While oxygen is typically used for this purpose,^[^ ^33^ ^]^ oxygen can potentially cause material decomposition through photo‐oxidation after photoexcitation of organic emitters with highly twisted bonds, which widely exist in TADF materials.^[^ ^37^ ^]^ Furthermore, oxygen may also quench singlet excitons.^[^ ^38^ ^]^ When considering PL measurements in film, oxygen quenching may be incomplete due to slow oxygen diffusion.

Therefore, we have opted to use anthracene as an alternative triplet scavenger. A suitable triplet scavenger needs to keep the singlet dynamics unaffected, therefore the singlet level of the triplet quencher should be higher than that of the TADF emitter, to avoid singlet quenching. Furthermore, it should not accelerate the ISC process to prevent converting singlets into triplet excitations. Finally, when CzDBA molecules are excited, the triplet quencher should not be excited simultaneously. These requirements are met by anthracene. The chemical structure and energy levels are shown in Figures S2 and S4 (Supporting Information), respectively. Since the singlet energy of anthracene is ∼3.1 eV, much higher than that of CzDBA (∼2.63 eV), singlet energy transfer from CzDBA to anthracene is avoided.^[^ ^39^ ^]^ When excited at 400 nm, only CzDBA is excited, while anthracene is not absorptive, as shown in Figure S5 (Supporting Information).^[^ ^31^ ^]^

With anthracene as the triplet scavenger, a clear PL emission from CzDBA can be observed, with the intensity decay shown as blue symbols in Figure 4, while the time‐dependent spectra are shown in Figure S6 (Supporting Information). The PL decay is manifested as a monoexponential decay, closely following the prompt part of the PL trace in degassed solution. Moreover, as shown in Figure 4, the theoretical prediction (blue dashed line) is nearly identical to the measured decay. Therefore, for TADF emitters, the lifetime of residual PL decay with complete triplet quenching can also be explicitly predicted from the biexponential decay for the TADF system in an inert atmosphere. Since both the PL lifetime and quantum efficiency with complete triplet quenching follow directly from measurements in inert atmosphere, triplet quenching measurements are redundant and thus cannot be used to resolve nonradiative decay channels in a given TADF system.

### 2.4 Absolute Verification for TADF Emitters with Non‐Unity PLQY

When a TADF emitter is used in an OLED, the origin of nonradiative losses has a strong effect on the device efficiency, since 75% of the generated excitons are in the triplet state. This effect is captured in the charge‐to‐photon efficiency *η*_CTP_, describing the quantum yield of emissive excitons under electrical excitation. The charge‐to‐photon efficiency can be expressed in terms of kinetic rates (Equation S8c, Supporting Information), but shows a simple relation with the PLQY and the ratio parameter *α* (i.e., Equation 7):

(13) $$\eta_{\mathrm{CTP}}=\frac{1+3\alpha }{4}\;\eta_{\mathrm{PLQY}}\;$$

As a result, in the absence of nonradiative triplet decay, i.e., *k*_T_ = 0 and thus *α* = 1, the efficiency *η*_CTP_ reaches its maximum value, being equal to *η*_PLQY_. In the opposite case that all losses are triplet related *η*_CTP_ can be approximated by Equation S8d (Supporting Information).

Hence, in the two extreme cases of losses either fully due to singlets or triplets, the OLED efficiency can already be predicted from the biexponential fit parameters and the PLQY. For in‐between cases, it is obvious that *η*_CTP_ will be smaller than η_PLQY_. Furthermore, Equation 13 shows that the *η*_CTP_ is monotonically dependent on the parameter *α*. Therefore, once *η*_CTP_ is determined individually from OLED analysis, one can determine the parameter α unambiguously, and then the rest of key parameters for exciton dynamics from the transient PL decay and PLQY (i.e., Equation (7), (8), (9) and Equation S4, Supporting Information). In the following section, we present a method to quantify *η*_CTP_ from OLED analysis, which allows for quantification of all kinetic rates when combined with PL experiments.

For TADF‐OLEDs without exciton annihilation, the external quantum efficiency (EQE) is determined by the electrical efficiency *γ*, the charge‐to‐photon efficiency *η*_CTP_ and optical outcoupling efficiency *η*_out_:^[^ ^40^ ^]^

(14) $$EQE=\gamma \cdot \eta_{\mathrm{CTP}}\cdot \eta_{\mathrm{out}}$$

**Figure 5:**
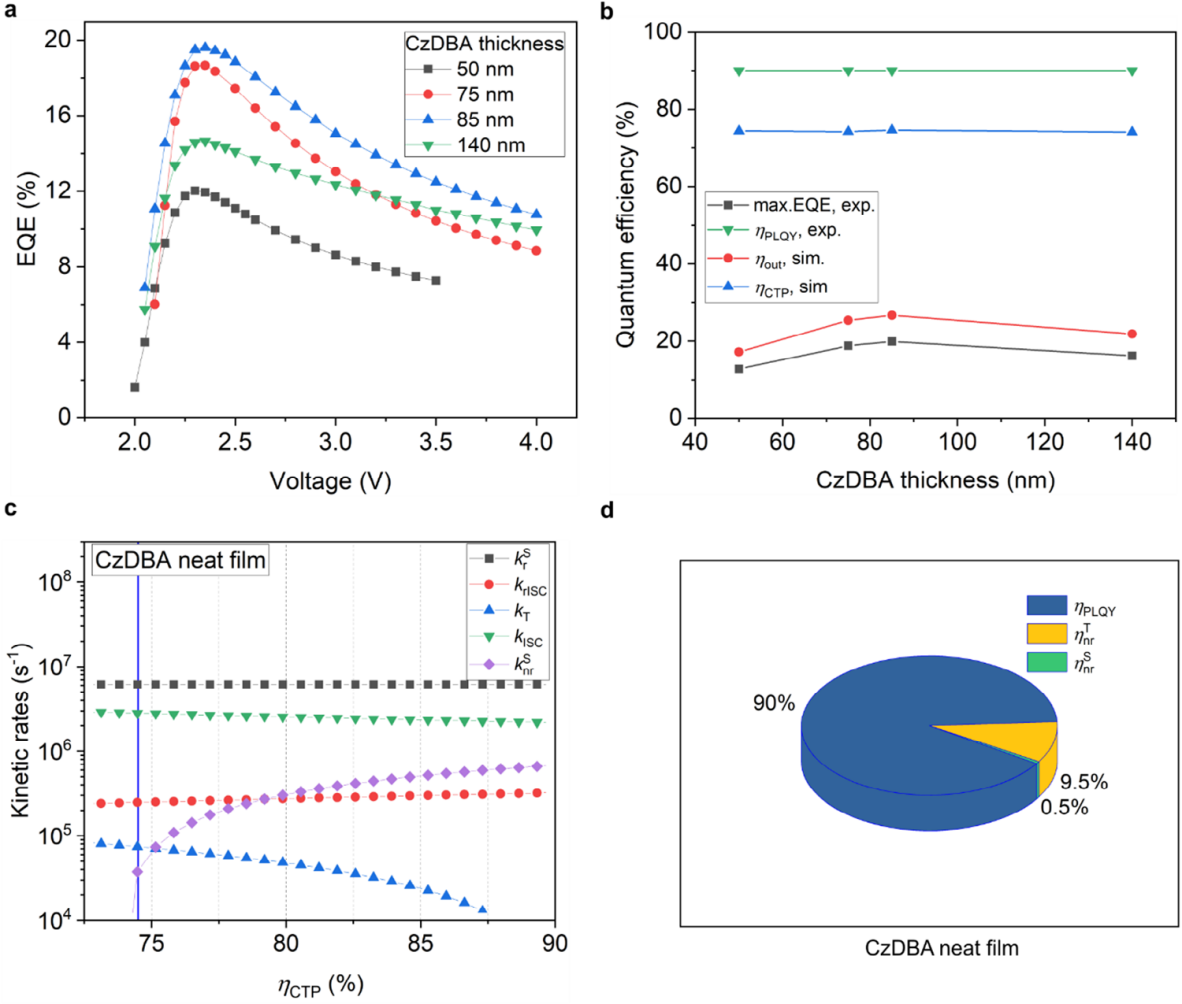
The verification of kinetic rates with OLEDs performance. a) The voltage dependent EQE for OLEDs with neat CzDBA at different thicknesses. b) The calculated CTP efficiency from the experimental EQE and simulated η_out_. c) Absolute verification of kinetic rates with the CTP efficiency from devices (blue line). d) The quantum efficiency for PLQY, losses from singlets ($\eta_{\mathrm{nr}}^{S}$) and triplets ($\eta_{\mathrm{nr}}^{T}$) for CzDBA neat film under PL excitation.

To properly determine *η*_CTP_ from device experiments, one needs to know the electrical efficiency *γ* and optical outcoupling efficiency *η*_out_. Here, we choose the benchmark CzDBA OLEDs to demonstrate the concept, since charge transport and efficiency roll‐off have been systematically investigated previously.^[^ ^7^, ^41^, ^42^ ^]^ In the single‐layer CzDBA OLEDs, a neat layer of CzDBA is sandwiched between an Ohmic anode and cathode. The devices exhibit balanced transport, with high EQEs at low driving voltages where the exciton annihilation has minor impact on the device efficiency, and long operational lifetime.^[^ ^26^, ^30^, ^32^ ^]^ The electrical characteristics and OLED efficiency are shown in Figure S6 (Supporting Information). Because *η*_CTP_ describes the quantum yield of emissive excitons under electrical excitation, it is independent of the electrical transport or optical outcoupling of devices. Therefore, the modeled *η*_CTP_ of OLEDs with different CzDBA layer thicknesses should in principle be equal. To confirm this deduction and therefore enhance the reliability of the modeled *η*_CTP_, we calculate the *η*_CTP_ for OLEDs with several thicknesses, i.e., with different optical outcoupling efficiency *η*_out_. The experimentally determined EQE as a function of driving voltages is shown in **Figure** **5**a. These devices reach a maximum EQE in the range of 12–19% at only ∼2.2–2.4 V, due to efficient charge injection with two Ohmic contacts and a high electron and hole mobility.^[^ ^28^ ^]^

In single‐layer OLEDs, the recombination occurs positionally dependent throughout the emissive layer and the position of the recombination zone solely depends on the charge transport in the layer. Since there are no blocking layers to confine the recombination zone, charge and exciton accumulation is prevented, minimizing annihilation effects. With the charge‐transport independently obtained from hole‐only and electron‐only devices, the shape and position of the recombination zone can be simulated with a drift‐diffusion model.^[^ ^4^, ^43^ ^]^ We have previously demonstrated that the optical outcoupling efficiency is weighted by the recombination profile at each position, for devices with a broad recombination zone.^[^ ^29^ ^]^ With the recombination profile known for the single‐layer CzDBA OLEDs, we can simulate *η*_out_ at the voltages at which EQE reaches its maximum value, presented in Figure 5b, which is 17–27% depending on the CzDBA layer thickness. In the current work, the Purcell effect is not considered, because the resonance effect of OLEDs investigated is relatively weak. For OLEDs with stronger cavity effects such as top‐emitting OLEDs, the Purcell effect might be important since it can alter the singlet radiative rate.^[^ ^40^, ^44^, ^45^, ^46^ ^]^

Since the single‐layer OLEDs have ohmic electron and hole contacts, all charges must recombine within the emissive layer.^[^ ^47^ ^]^ Due to the high density of electrons and holes near the ohmic contacts, charges cannot traverse the emissive layer without recombining, therefore the electrical efficiency *γ* for these devices is 1. We have recently shown that recombination from the small amount of traps in CzDBA OLEDs is emissive, while the trap density of only 1–2 × 10^22^ m^−3^ is filled at the voltage at which the maximum EQE is obtained.^[^ ^27^ ^]^ Indeed, as shown in Figure S7 (Supporting Information), there is only minor spectral difference between PL and EL for the CzDBA neat film, which might be an indication that the emissive traps are from dimers.^[^ ^32^, ^48^ ^]^ Moreover, the difference of EL spectra in CzDBA OLEDs with different cavity lengths might stem from the resonance effect.^[^ ^29^ ^]^ Since the emissive trap density is much lower compared to the molecular density in the range of 10^27^ m^−3^, the simplification with the three‐level model for the PL decay is acceptable for the CzDBA system, considering the biexponential PL decay (Figure 4a) as a signature of three‐level systems.

Based on the modeled *η*_out_ and Equation 14, we can finally calculate *η*_CTP_ for all these devices from the experimentally measured EQEs, shown in Figure 5b. The efficiency *η*_CTP_ is estimated to be 74–75%, with only a minor deviation from different devices. Moreover, *η*_CTP_ is lower than the PLQY of a CzDBA neat film, which is 90%, indicating the presence of triplet nonradiative losses.

Based on the PLQY of 90% and the biexponential fitting parameters shown in Figure 2c, the possible combination of kinetic rates as a function of *η*_CTP_ is shown in Figure 5c. Combined with the obtained efficiency *η*_CTP_ from devices, we can then determine the unique combination of kinetic rates. The summary of the full photophysical properties of the CzDBA neat film is shown in **Table** **2**.

**Table 2:** Photophysical parameters for CzDBA neat film.

*η*_PLQY_	A_1_	A_2_	τ_PF_ [ns]	τ_DF_ [µs]	*k*_S_ [s^−1^]	*k*_ISC_ [s^−1^]	*k*_rISC_ [s^−1^]	$k_{r}^{S}$[s^−1^]	$k_{\mathrm{nr}}^{S}$[s^−1^]	*k*_T_ [s^−1^]	*η*_CTP_
90.0%	0.991	0.009	110	4.12	6.2 × 10^6^	2.8 × 10^6^	2.5 × 10^5^	6.2 × 10^6^	3.7 × 10^4^	7.4 × 10^4^	74.5%

With the determined kinetic rates for CzDBA neat film, based on Equation S5 (Supporting Information), we can further calculate the nonradiative losses from triplets under PL excitation. As shown in Figure 5d, for CzDBA neat film, the major nonradiative loss is due to nonradiative relaxations from triplets (9.5%). The rest of losses from singlets only make a minor contribution (0.5%). For such an efficient TADF system with a PLQY as high as 90%, the triplet nonradiative loss is still the major channel.

Figure 5c shows that attributing nonradiative losses to either singlets or triplets can lead to significant errors in the determination of the kinetic rates in TADF systems. We note that eventual inaccuracies in the measurements and fitting of the experimental decay curves will be largely overruled by the lack of knowledge on the origin of the nonradiative recombination, which can lead to variations in the derived kinetic rates as large as an order of magnitude. This then in turn hinders the understanding of exciton dynamics and can lead to erroneous conclusions regarding the expected performance in TADF‐based OLEDs. Moreover, since the triplet nonradiative loss plays an important role in the OLED efficiency, an exact quantification is indispensable for understanding its molecular origin and further development of novel TADF emitters.

Our experimentally determined reversed intersystem crossing rate *k*_rISC_ can also be compared with predictions from Marcus theory and first‐principles that have been used to simulate the energy levels and kinetic rates of some TADF emitters. Specifically, 4CzIPN as a benchmark emitter, the simulated *k*_rISC_ rate has been investigated for different groups. Shuai's group showed that the simulated *k*_rISC_ rate is 1.23 × 10^6^ s^−1^ at 300 K, with the simulated singlet radiative rate as 1.11 × 10^7^ s^−1^ and the nonradiative rate of 2.37 × 10^5^ s^−1^.^[^ ^49^ ^]^ Furthermore, in a different publication by Brédas et. al., the simulated *k*_rISC_ rate of 4CzIPN is 4.92 × 10^6^ s^−1^.^[^ ^50^ ^]^ Comparing our results for 4CzIPN in toluene at room temperature, the experimentally determined *k*_rISC_ rate of 1.23 × 10^6^ s^−1^ is very comparable to that predicted by Shuai's group and 4 times lower than that predicted by Brédas’ group. The singlet radiative rate is also within the same order of magnetite of that predicated by Shuai's group. Therefore, the experimental result is comparable to the simulated values.

The method proposed can be universally used for various TADF systems with biexponential PL decays, with different emissive spectra and PLQYs. We note that our model system CzDBA in the sold state exhibits a high PLQY of 90%. In spite of the high PLQY, as shown in Figure 5c, the uncertainty in obtained kinetic rates is still large, depending on the origin of the nonradiative losses. A question is whether our approach is also valid for systems with a lower PLQY, as for example NIR emitters with strong nonradiative losses (either from singlets or triplets) as a result of the energy‐gap law. In a recent study, we investigated the optical properties of the blue‐emitting TADF material 9,9′,9′‐(5‐(4,6‐diphenyl‐1,3,5‐triazin‐2‐yl)benzene‐1,2,3‐triyl)tris (9H‐carbazole) (3CzTRZ).^[^ ^51^ ^]^ Neat films of 3CzTRZ exhibit a low photoluminescence quantum yield (PLQY) of only 40%, resulting in OLEDs with a low EQE of 4.5%. By diluting 3CzTRZ in a large bandgap host, the triplet lifetime is increased by at least an order of magnitude, resulting in an enhanced PLQY of 70% and a more than three‐fold enhancement of the EQE to 15% in a single‐layer blue OLED. The measured OLED efficiencies of the pristine and diluted system then can again be used to determine the intrinsic kinetic rates of the 3CzTRZ emitter, as shown in Figure S8 (Supporting Information). Experimentally, we observed a huge difference between the PL properties in solutions and in films with and without a host. Our results demonstrate that the molecular environment plays a central role in the exciton kinetics of TADF systems, and therefore the device performance. Further understanding of the origin of nonradiative losses in TADF emitters is therefore a prerequisite for the realization of efficient TADF‐based OLEDs.

## 3 Conclusion

In conclusion, we have demonstrated that the kinetic rates of excitonic processes in TADF emitters cannot be uniquely determined based on transient PL and PLQY measurements alone. An exception is the case of emitters with unity PLQY, for which we demonstrate simple equations for obtaining the kinetic rates from a PL decay. For emitters with non‐unity PLQY, it is unknown whether losses are due to nonradiative singlet decay, which prevents unambiguous determination of the kinetic rates. We demonstrate that additional measurements with triplet quenchers are not able to resolve this issue. The reason is that transient PL measurements in inert atmosphere can already predict the PL decay and PLQY in the presence of triplet quenchers. In order to finally obtain unique values for the kinetic rates, PL experiments are combined with the charge‐to‐photon efficiency evaluated from OLED measurements. For CzDBA neat films with a PLQY as high as 90%, it is found that the nonradiative loss mainly results from triplets. We anticipate that this work contributes to an improved interpretation of exciton dynamics in TADF emitters.

## 4 Experimental Section

### Device Fabrication

ITO substrates were cleaned in an ultrasonic bath for 5 min in acetone first and then in isopropyl alcohol. These cleaned ITO substrates were then dried in an heating oven at 140 °C and treated by UV‐ozone for another 20 min. Afterward, a layer of PEDOT: PSS (AI4083, 40 nm) was coated and then annealed on a hotplate at 140 °C. The following functional layers including MoO_3_ (6 nm), C_60_ (3 nm), CzDBA (50, 75, 85, or 140 nm), 2,2′,2′'‐(1,3,5‐Benzinetriyl)‐tris(1‐phenyl‐1‐H‐benzimidazole) (TPBi, 4 nm) and Al (∼ 100 nm) were sequentially deposited in a vacuum evaporator with a pressure of 4–6 × 10^−7^ mbar. The current density‐voltage characteristics were measured by a Keithley 2400 SMU, with the electroluminescence measured by a calibrated Si photodiode with a larger area than the emitting pixel. The electroluminescent spectra were measured by a USB4000‐UV–vis–ES spectrometer.

### Transient PL Measurement

The detailed settings of the transient PL measurements can be found in the footnote of figures.

### Optical Simulation

The optical parameters such as optical constants and dipole orientation factors for CzDBA neat film has been experimentally measured and published previously.^[^ ^29^ ^]^

## Conflict of Interest

The authors declare no conflict of interest.

## Supporting information



Supporting Information

## Data Availability

The data that support the findings of this study are available from the corresponding author upon reasonable request.
