# Supplementary material for: Determination of the Kinetic Rates and Intrinsic Nonradiative Losses in Organic Thermally Activated Delayed Fluorescence Emitters
Source: Adv Sci (Weinh). 2025 Jun 26;12(35):e05338. doi: 10.1002/advs.202505338 (PMC12463001; doi:10.1002/advs.202505338)
Supplement: Supplementary file 1 — Supporting Information [file ADVS-12-e05338-s001.docx]

Supporting Information

**Determination of the kinetic rates in organic thermally activated delayed fluorescence emitters**

*Xin Zhou, Jawid Nikan, Kaiwen Guo, Paul W. M. Blom*, Gert-Jan A. H. Wetzelaer* and Yungui Li**

Under the transient case, singlet *n*_S_ and triplet *n*_T_ densities for TADF emitters with a three-level model can be described as:

$\frac{dn_{S}}{dt}=-k_{S}n_{S}-k_{ISC}n_{S}+k_{rISC}n_{T}$, (Eq. S1a)

$\frac{dn_{T}}{dt}=k_{ISC}n_{S}-k_{rISC}n_{T}-k_{T}n_{T}$. (Eq. S1b)

in which *k*_s_ = $k_{r}^{S}$+$k_{nr}^{S}$, with $k_{r}^{S}$ as the singlet radiative rate and $k_{nr}^{S}$ the singlet nonradiative rate. In the above equation, *k*_ISC_ is the ISC rate, *k*_rISC_ the rISC rate and *k*_T_ the triplet recombination rate.

**Under PL excitation in an inert atmosphere**

Under the PL excitation, with the initial condition *n*_S_= *n*_0_ and *n*_T_=0, the analytical solutions for Eq. S1 write as following:

$n_{S}=\frac{n_{0}\left( \lambda_{1}{+k}_{rISC}+k_{T} \right)}{\lambda_{1}-\lambda_{2}}e^{\lambda_{1}t}-\frac{n_{0}\left( \lambda_{2}+k_{rISC}+k_{T} \right)}{\lambda_{1}-\lambda_{2}}e^{\lambda_{2}t}$, (Eq. S2a)

$n_{T}= \frac{k_{ISC}n_{0}}{\lambda_{1}-\lambda_{2}}\left( e^{\lambda_{1}t}-e^{\lambda_{2}t} \right)$, (Eq. S2b)

$\lambda_{1}=\frac{-\left( k_{S}+k_{ISC}+k_{rISC}+k_{T} \right)+\sqrt{\left( k_{S}+k_{ISC}-k_{rISC}-k_{T} \right)^{2}+4k_{ISC}k_{rISC}}}{2}$, (Eq. S2c)

$\lambda_{2}=\frac{-\left( k_{S}+k_{ISC}+k_{rISC}+k_{T} \right)-\sqrt{\left( k_{S}+k_{ISC}-k_{rISC}-k_{T} \right)^{2}+4k_{ISC}k_{rISC}}}{2}$, (Eq. S2d)

$\lambda_{1}-\lambda_{2}=\sqrt{\left( \left( k_{S}+k_{\mathrm{ISC}} \right)-\left( k_{\mathrm{rISC}}+k_{T} \right) \right)^{2}+4k_{\mathrm{ISC}}k_{\mathrm{rISC}}}$. (Eq. S2e)

The PLQY in an inert atmosphere can be determined as:

$\eta_{\mathrm{PLQY}}=\frac{1}{n_{0}}\int_{0}^{\infty} {k_{r}^{S}n}_{S}dt=\frac{k_{r}^{S}\left( k_{rISC}+k_{T} \right)}{\lambda_{1}\lambda_{2}}$ . (Eq. S3a)

The triplet quantum loss from triplet nonradiative recombination can be determined as:

$\eta_{nr}^{T}=\frac{1}{n_{0}}\int_{0}^{\infty} {k_{T}n}_{T}dt=\frac{{k_{T}k}_{ISC}}{\lambda_{1}\lambda_{2}}$ . (Eq. S3b)

**Optical quantities described by biexponential parameters**

When the PL decay in an inert atmosphere is fitted with a biexponential decay A_1_exp(-t/τ_PF_)+A_2_exp(-t/τ_DF_), the fitted lifetimes in an inert atmosphere is kinetically determined as:

$\tau_{\mathrm{PF}}=\frac{1}{{-\lambda}_{2}}$, (Eq. S4a)

$\tau_{\mathrm{DF}}=\frac{1}{{-\lambda}_{1}}$. (Eq. S4b)

With the defined parameter *α*, the kinetic rates *k*_T_ can be determined as:

$k_{T}=(1-\alpha)\left( \frac{A_{2}}{\tau_{PF}}+\frac{A_{1}}{\tau_{\mathrm{DF}}} \right)$. (Eq. S4c)

Therefore, based on the transient PL and PLQY results, we can finally determine:

$k_{S}=\frac{A_{1}}{\tau_{\mathrm{PF}}}+\frac{A2}{\tau_{\mathrm{DF}}}-k_{\mathrm{ISC}}=\frac{A_{1}}{\tau_{\mathrm{PF}}}+\frac{A2}{\tau_{\mathrm{DF}}}-\frac{1}{\alpha}\frac{{\frac{\tau_{\mathrm{PF}}}{\tau_{\mathrm{DF}}}\left( \frac{\tau_{\mathrm{DF}}}{\tau_{\mathrm{PF}}}-1 \right)}^{2}}{\frac{\tau_{\mathrm{DF}}}{A1}+\frac{\tau_{\mathrm{PF}}}{A2}}$, (Eq. S4d)

$k_{\mathrm{nr}}^{S}=k_{S}-k_{r}^{S}=\frac{A_{1}}{\tau_{\mathrm{PF}}}+\frac{A2}{\tau_{\mathrm{DF}}}-\frac{1}{\alpha}\frac{{\frac{\tau_{\mathrm{PF}}}{\tau_{\mathrm{DF}}}\left( \frac{\tau_{\mathrm{DF}}}{\tau_{\mathrm{PF}}}-1 \right)}^{2}}{\frac{\tau_{\mathrm{DF}}}{A1}+\frac{\tau_{\mathrm{PF}}}{A2}}-\frac{\eta_{\mathrm{PLQY}}}{{A_{1}\tau}_{PF}+A_{2}\tau_{DF}}$. (Eq. S4e)

The nonradiative loss from triplets $\eta_{\mathrm{nr}}^{T}$ intrinsically described as Eq. S3b, can also be described with the experimental parameters and *α*:

$\eta_{\mathrm{nr}}^{T}=A_{1}A_{2}\frac{1-\alpha}{\alpha}{\frac{\tau_{\mathrm{PF}}}{\tau_{\mathrm{DF}}}\left( \frac{\tau_{\mathrm{DF}}}{\tau_{\mathrm{PF}}}-1 \right)}^{2}$. (Eq. S5)

**PL with complete triplet quenching**

When assuming efficient and complete triplet quenching in oxygen-rich atmosphere without photo-oxidization, the PL decay is only determined by the singlet decay including the spontaneous relaxation to the ground state with a rate of *k*_S_ and the ISC process with a rate of *k*_ISC_. Therefore, the transient singlet decay for TADF emitters with complete triplet quenching can be described as:

$n_{S}= n_{0}e^{-\left( k_{S}+k_{\mathrm{ISC}} \right)t}$*,* (Eq. S6a)

where *n*_0_ is the initial singlet density. In other words, theoretically, the singlet decay in oxygen is monoexponential. As a result, the PLQY of the residual fluorescence $\eta_{\mathrm{PF}}^{*}$ with perfect triplet quenching is determined as:

$\eta_{\mathrm{PF}}^{*}=\frac{1}{n_{0}}\int_{0}^{\infty} {k_{r}^{S}n}_{S}dt=\frac{k_{r}^{S}}{k_{S}+k_{\mathrm{ISC}}}$, (Eq. S6b)

with a lifetime:

$\tau_{\mathrm{PF}}^{*}=\frac{1}{k_{S}+k_{\mathrm{ISC}}}$. (Eq. S6c)

The quenched fluorescence is then the difference between the PLQY *η*_PLQY_ in an inert atmosphere and the residual fluorescence $\eta_{\mathrm{PF}}^{*}$ with complete triplet quenching:

$\eta_{\mathrm{DF}}^{*}=\eta_{\mathrm{PLQY}}-\eta_{\mathrm{PF}}^{*}$. (Eq. S6d)

Alternatively, the quantum yield of quenched fluorescence with complete triplet quenching, previously originating from the rISC process in an inert atmosphere, can be derived as:

$\eta_{\mathrm{DF}}^{*}=\frac{1}{n_{0}}\int_{0}^{\infty} \frac{k_{r}^{S}}{k_{S}+k_{\mathrm{ISC}}}{k_{\mathrm{rISC}}n}_{T}dt=\frac{k_{r}^{S}}{k_{S}+k_{\mathrm{ISC}}}k_{\mathrm{ISC}}k_{\mathrm{rISC}}\frac{1}{\lambda_{1}\lambda_{2}}$. (Eq. S6e)

Therefore, for TADF emitters, the ratio between the quantum yield of quenched DF and the remaining PF part is intrinsically related with its kinetic rates as:

$\frac{\eta_{\mathrm{DF}}^{*}}{\eta_{\mathrm{PF}}^{*}}=\frac{k_{\mathrm{ISC}}k_{\mathrm{rISC}}}{\lambda_{1}\lambda_{2}}$. (Eq. S6f)

Furthermore, one can predict the quenched DF with triplet scavengers, with experimental results obtained in nitrogen explicitly:

$\eta_{\mathrm{DF}}^{*}=\frac{A_{1}A_{2}\eta_{\mathrm{PLQY}}\left( \frac{{\tau_{DF}-\tau}_{PF}}{\tau_{PF}\tau_{DF}} \right)^{2}}{\left( \frac{A_{2}}{\tau_{PF}}+\frac{A_{1}}{\tau_{\mathrm{DF}}} \right)\left( \frac{A_{1}}{\tau_{PF}}+\frac{A_{2}}{\tau_{\mathrm{DF}}} \right)}$. (Eq. S7a)

The ratio of quantum yields between the quenched DF and the remaining PF can also be described explicitly using the bi-exponential fitting parameters of the transient PL decay:

$\frac{\eta_{\mathrm{DF}}^{*}}{\eta_{\mathrm{PF}}^{*}}={A_{1}A}_{2}{\frac{\tau_{\mathrm{PF}}}{\tau_{\mathrm{DF}}}\left( \frac{\tau_{\mathrm{DF}}}{\tau_{\mathrm{PF}}}-1 \right)}^{2}$. (Eq. S7b)

**Under transient EL excitation in an inert atmosphere**

Under the EL excitation in devices with the initial condition *n*_S_= *N*_0_/4 and *n*_T_=3*N*_0_/4 for Eq. S1, in which *N*_0_ is the total number of recombined excitons, we can then obtain the analytical description for singlet and triplet densities as a function of time:

$n_{S}^{EL}=\frac{N_{0}\left( \lambda_{1}{+k}_{rISC}+k_{T} \right)\left( 3\lambda_{2}-k_{ISC}+{3k}_{rISC}+3k_{T} \right)}{4\left( \lambda_{2}-\lambda_{1} \right)k_{ISC}}e^{\lambda_{1}t}+\frac{N_{0}\left( \lambda_{2}{+k}_{rISC}+k_{T} \right)\left( k_{ISC}-3\lambda_{1}{-3k}_{rISC}-3k_{T} \right)}{4\left( \lambda_{2}-\lambda_{1} \right)k_{ISC}}e^{\lambda_{2}t}$, (Eq. S8a)

$n_{T}^{EL}=\frac{N_{0}\left( 3\lambda_{2}-k_{ISC}+{3k}_{rISC}+3k_{T} \right)}{4\left( \lambda_{2}-\lambda_{1} \right)}e^{\lambda_{1}t}+\frac{N_{0}\left( k_{ISC}-3\lambda_{1}{-3k}_{rISC}-3k_{T} \right)}{4\left( \lambda_{2}-\lambda_{1} \right)}e^{\lambda_{2}t}$. (Eq. S8b)

Therefore, the analytical description for the CTP efficiency $\eta_{ctp}$ based on TADF emission, can be described as:

$$\eta_{ctp}=\frac{1}{N_{0}}\int_{0}^{\infty} k_{r}^{S}n_{S}^{EL}dt$$

$=k_{r}^{S}\frac{\left( \lambda_{1}{+k}_{rISC}+k_{T} \right)\left( 3\lambda_{2}-k_{ISC}+{3k}_{rISC}+3k_{T} \right)}{4\left( {\lambda_{1}-\lambda}_{2} \right)k_{ISC}\lambda_{1}}+k_{r}^{S}\frac{\left( \lambda_{2}{+k}_{rISC}+k_{T} \right)\left( k_{ISC}-3\lambda_{1}{-3k}_{rISC}-3k_{T} \right)}{4\left( {\lambda_{1}-\lambda}_{2} \right)k_{ISC}\lambda_{2}}.$ (Eq. S8c)

In the case that all losses are triplet related, *η*_CTP_ can be approximated by

$\eta_{CTP}\cong\frac{\eta_{PLQY}}{4}+\frac{\frac{3}{4}\eta_{PLQY}}{\left( 1-\eta_{PLQY} \right)\frac{\tau_{\mathrm{PF}}}{{A_{1}A}_{2}\tau_{\mathrm{DF}}}+\text{1 }}$ (Eq. S8d)

(a) (b)

Figure S1. Molecular structure of (a) CzDBA and (b) anthracene.


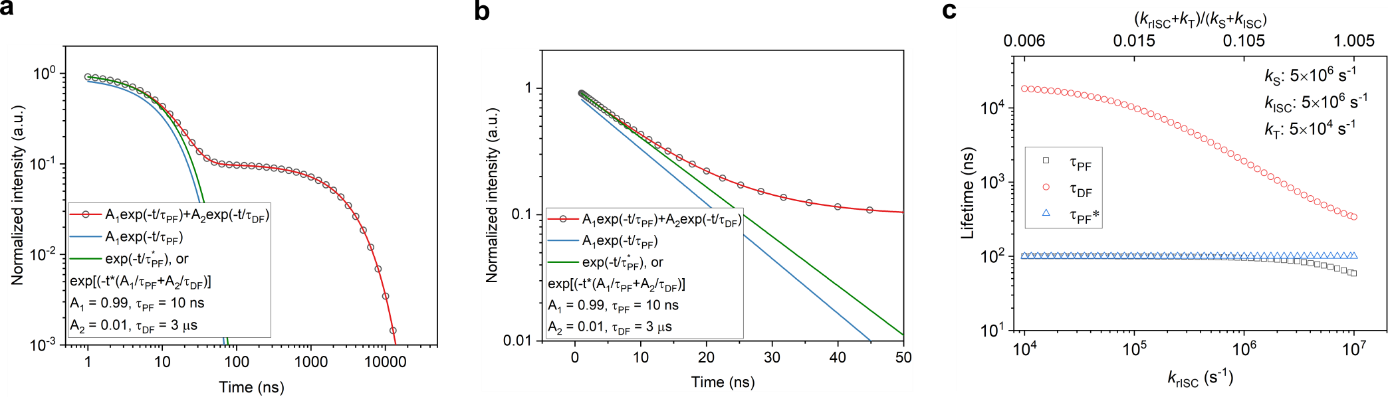


Figure S2. Direct determination of the ${}_{\mathrm{PF}}^{*}$ in the transient PL in inert atmosphere in a lin(t)-log(I) plot (a) and (b) a zoom-in plot. (c)The comparision of τ_PF_, ${}_{\mathrm{PF}}^{*}$ and τ_DF_ for PL decays with a scan of *k*_rISC_ rates.

As ${}_{\mathrm{PF}}^{*}$ coincides with the time constant for the prompt part of the PL decay in inert atmosphere (Figure S2a and S2b), one may wonder how it differs from $\tau_{\mathrm{PF}}$. To this end, it can be demonstrated that the slope at time zero on a semi-logarithmic plot of a biexponential PL decay in inert atmosphere explicitly determines ${}_{\mathrm{PF}}^{*}$, in a plot with the natural logarithmic intensity as the y-axis and a linear time as the x-axis. For a biexponential PL decay, described as *I* = *A*_1_exp(-*t*/*τ*_PF_)+*A*_2_exp(-*t*/*τ*_DF_), the slope of ln(*I*) is time dependent, which is given by

$\frac{\mathrm{dln}(I)}{dt}=\frac{A_{1}}{-\tau_{PF}}\exp\left( -\frac{t}{\tau_{PF}} \right)+\frac{A_{2}}{-\tau_{DF}}\exp\left( -\frac{t}{\tau_{DF}} \right)$. (Eq. S9a)

Therefore, the slope of ln(*I*) at time zero

$\left. \frac{dln\left( I \right)}{dt} \right|_{t=0}=-\frac{A_{1}}{\tau_{PF}}-\frac{A_{2}}{\tau_{DF}}=-{}_{\mathrm{PF}}^{*}$ , (Eq. S9b)

exactly equal to the lifetime of the residual PL with complete triplet quenching. This is also graphically illustrated in Figure S2b.

A subtle point that is often overlooked is that ${}_{\mathrm{PF}}^{*}$ represents the intrinsic lifetime of PF, whereas $\tau_{\mathrm{PF}}$ does not. The reason originates from the biexponential fitting A_1_exp(-t/τ_PF_)+A_2_exp(-t/τ_DF_), with (A_1_+A_2_=1), of the PL decay in inert atmosphere. According to this biexponential model, at *t*=0 there is already a fraction A_2_ of delayed fluorescence present. In reality, at *t*=0 the PL only consists of PF, meaning that the biexponential model underestimates the PF at *t*=0. This issue is avoided when the prompt fluorescence in inert atmosphere is fitted with a monoexponential function, to prevent the abovementioned underestimation. Such a monoexponential fit then provides ${}_{\mathrm{PF}}^{*}$ directly, as shown by Figure S2b, which is the intrinsic lifetime of the PF.

One can further compare the difference between $\tau_{\mathrm{PF}}$ from a biexponential fitting of the PL decay in an inert atmosphere and ${}_{\mathrm{PF}}^{*}$ with complete triplet quenching as a function of *k*_rISC_. In inert atmosphere, *τ*_PF_ is also impacted by *k*_rISC_ and *k*_T_. In contrast, the residual PF lifetime ${}_{\mathrm{PF}}^{*}$ should be a constant, as it is independent of *k*_rISC_. As rISC is responsible for the delayed fraction of fluorescence, the PF should physically be independent of the rISC processes, as correctly captured by${}_{\mathrm{PF}}^{*}$. As shown in Figure S2c, visible difference between $\tau_{\mathrm{PF}}$ and ${}_{\mathrm{PF}}^{*}$ can be resolved when *k*_rISC_ is comparable or higher than the sum of *k*_S_ and *k*_ISC_. However, when *k*_rISC_ and *k*_T_ are significantly smaller than *k*_S_ and *k*_ISC_, the lifetime of the residual PL with perfect triplet quenching is then close to that in an inert atmosphere. This is the case for most of the reported TADF systems, where the rISC rate is much lower than *k*_S_. The reason is that singlet relaxation is a spin-allowed and energetically favorable process, whereas the rISC process is a spin-forbidden transition that requires thermal activation to facilitate the molecular geometry reorganization.

Figure S3. Scheme of energy levels for CzDBA and anthracene.


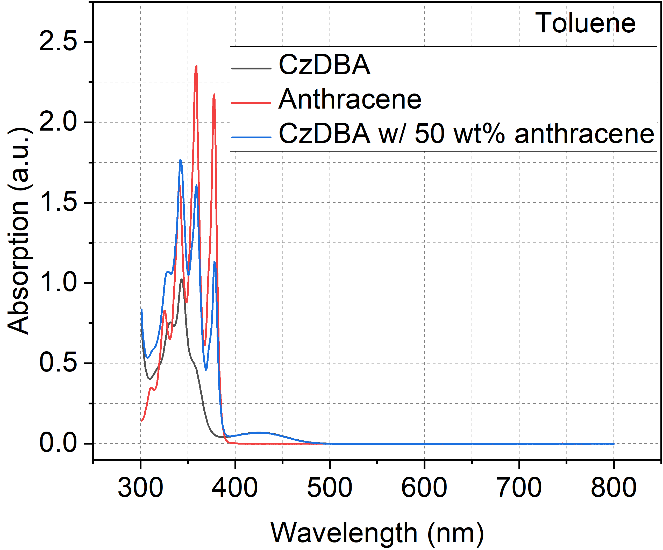


Figure S4. Absorption spectra for CzDBA, anthracene and their mixture in toluene.


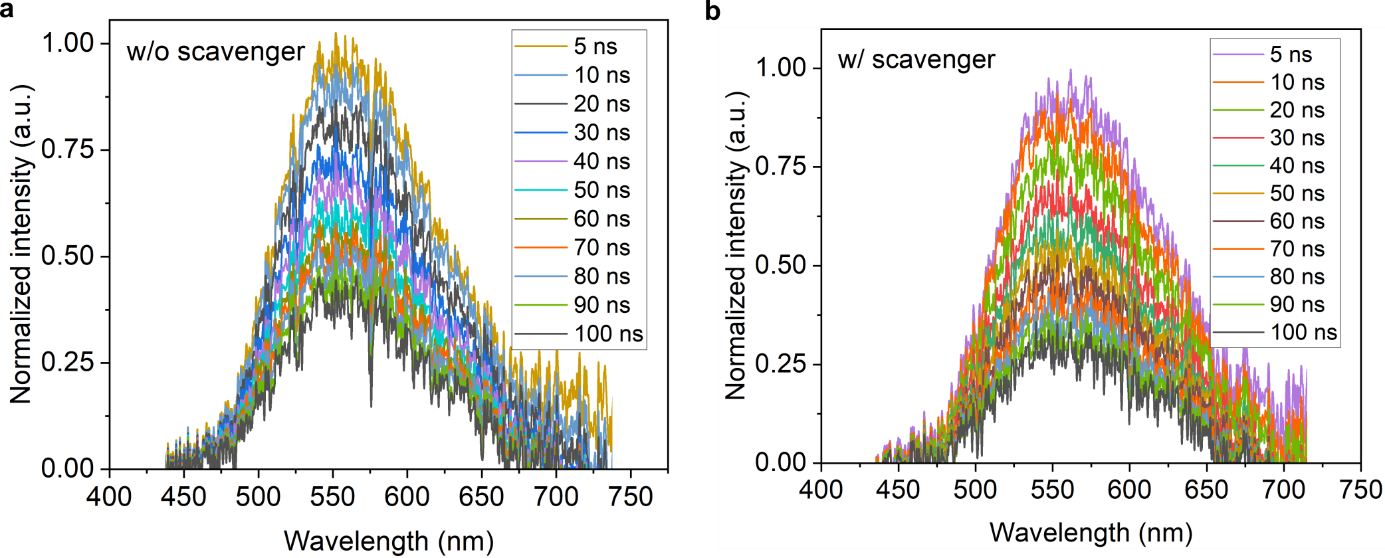


Figure S5. Transient PL spectra for CzDBA solution with or without triplet scavenger anthracene.


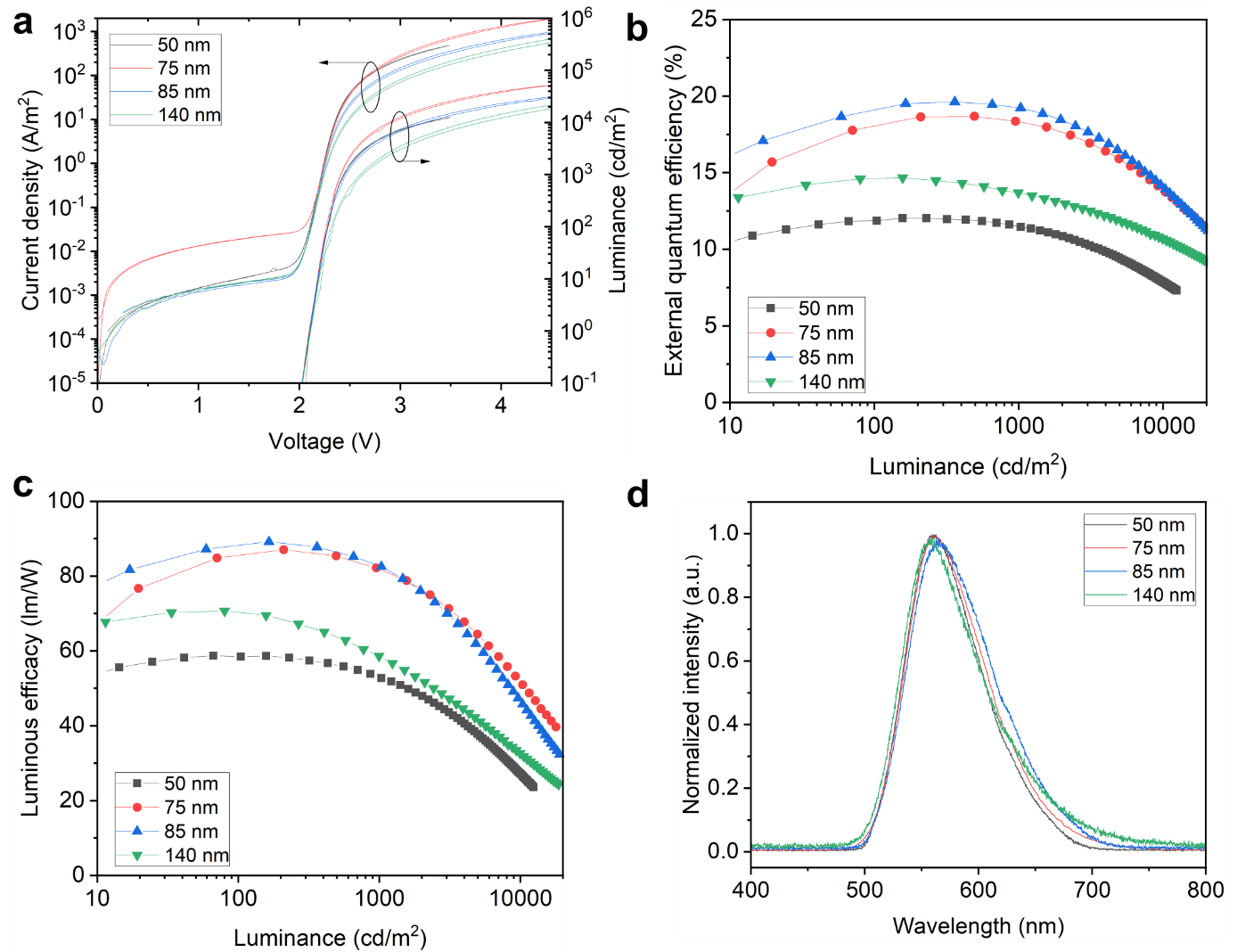


Figure S6. The device results for the single-layer CzDBA OLEDs with different cavity lengths. (a) JVL; (b) luminance-EQE; (c) luminance-luminous efficacy; (d) EL spectra. Taken from [ref: 10.1002/adom.202001812].

3. Details (procedures and results, etc.) may be provided in SI on the estimation of (i) the recombination zone profile in the single-layer OLED device and (ii) outcoupling efficiency.

The determination of recombination profiles and optical outcoupling efficiency for CzDBA OLEDs has been explained in detail in the reference paper [ref: 10.1002/adom.202001812]. Since it will be a duplication of presenting published results with a different focus (in the current work, we are focusing on the exciton dynamics of TADF systems), we therefore refer our previous work about these preliminary results on device optics.


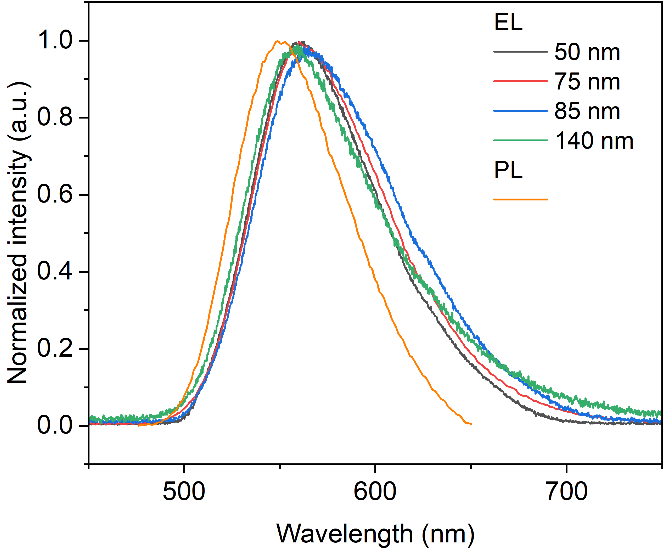


Figure S7. PL and EL spectra comparison for CzDBA neat film.


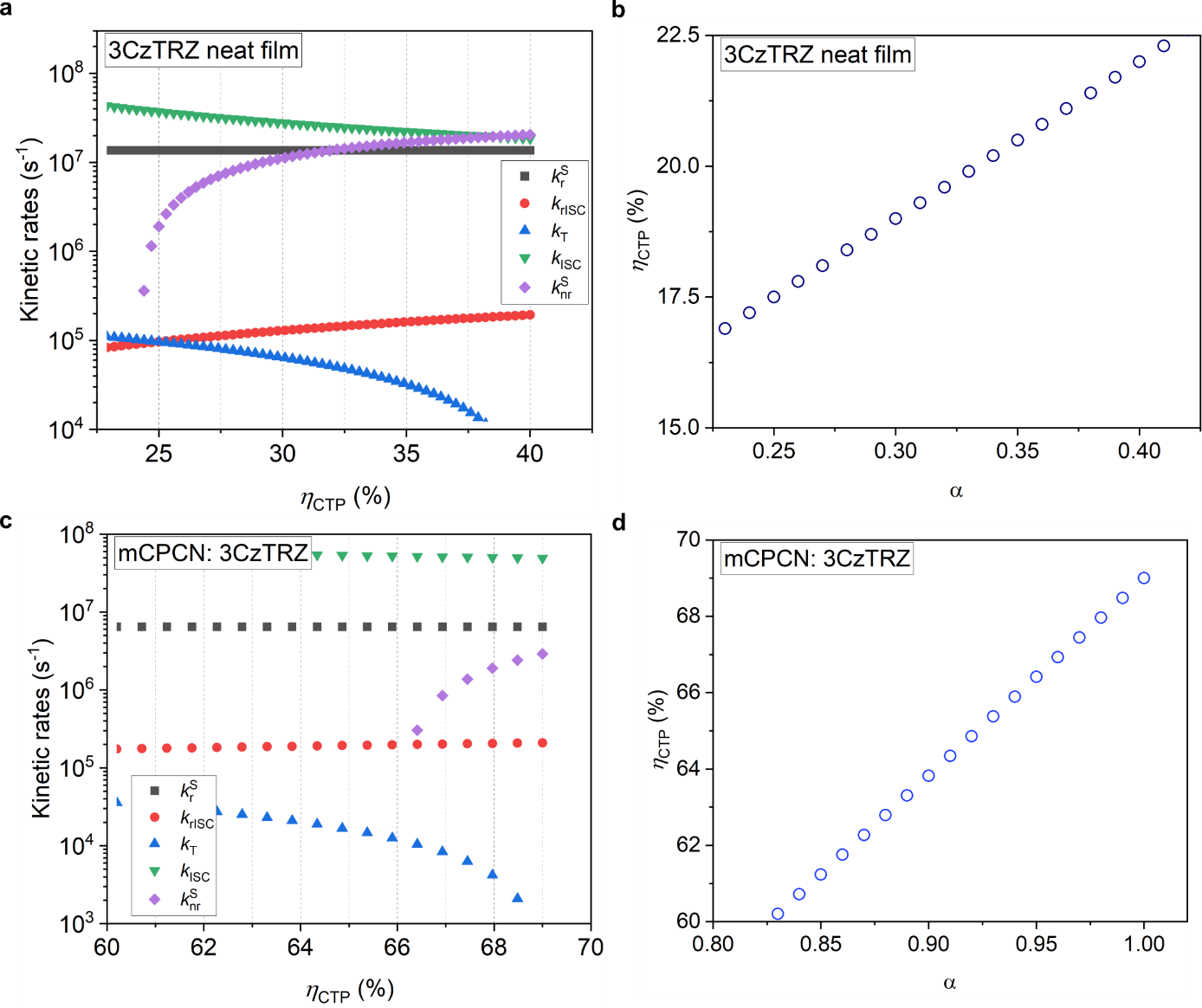


Figure S8. More examples to show the idea to determine the kinetic rates in other TADF systems with transient PL decay and device modelling. (a) Kinetic rates calculated with the derived analytical formula from the biexponential fitting parameters, and (b) charge-to-photon efficiency as a function of ratio parameter α for 3CzTRZ neat film. (c) Kinetic rates calculated with the derived analytical formula from the biexponential fitting parameters, and (b) charge-to-photon efficiency as a function of ratio parameter α for 3CzTRZ doped in mCPCN host. The EQE and optical outcoupling efficiency has been reported.^[1]^

To demonstrate that our approach also works for TADF systems with low-PLQY, we used a similar approach for two different blue TADF systems with much lower PLQYs (40% for 3CzTrz and 69% for 3CzTRz in an mCPCN host) to address the issue. The characterization of the charge transport and transient PL decay has been reported previously.^[1][2]^ In 3CzTRz the charge transport is perfectly balanced, whereas in the 3CzTRZ:mCPCN there is only a slight imbalance that does not strongly affect the operation of the OLED.

By fitting the transient PL decay^[1]^ with a biexponential function, we can then calculate the kinetic rates and charge-to-photon efficiency by the derived equations in the main text. Because the nonradiative loss from singlets and triplets cannot be determined yet, there is a possible range of charge-to-efficiency in the device. From the maximum device EQE and simulated outcoupling efficiency^[1]^, we can then determine the charge-to-photon efficiency by using Eq. 7 for these devices as well, as we did for CzDBA OLEDs in the manuscript. We determine a charge-to-photon efficiency of 25% for pristine 3CzTRZ and 65% for the 3CzTRZ:mCPCN blend. Using Figure S8a and S8c then allows us to precisely quantify the relevant kinetic rates. Therefore, the method can be generally used for different TADF systems with biexponential PL decay, with either high PLQY such as CzDBA or low PLQY such as 3CzTRZ neat films. It should be noted that there is bimolecular annihilation in the reported PL decay in 3CzTRZ systems.^[1]^ The proper determination of PL decay without annihilation needs future study, but this additional example shows that it is a general method to determine the kinetic rates from PL decays with biexponential fitting together with the device modelling.

References:

[1] O. Sachnik, X. Zhou, J. Nikan, B. van der Zee, Y. Li, P. W. M. Blom, G. A. H. Wetzelaer, *Adv. Opt. Mater.* **2024**, *12*, 1.

[2] O. Sachnik, X. Tan, D. Dou, C. Haese, N. Kinaret, K. H. Lin, D. Andrienko, M. Baumgarten, R. Graf, G. J. A. H. Wetzelaer, J. J. Michels, P. W. M. Blom, *Nat. Mater.* **2023**, *22*, 1114.
